# Challenges of extended venous thromboembolism prophylaxis in medical and surgical patients

**DOI:** 10.1590/1677-5449.202101951

**Published:** 2022-06-27

**Authors:** Maria Chiara Chindamo, Edison Ferreira Paiva, Plinio Resende do Carmo, Ana Thereza Cavalcanti Rocha, Marcos Arêas Marques

**Affiliations:** 1 Universidade Federal do Rio de Janeiro – UFRJ, Rio de Janeiro, RJ, Brasil.; 2 Hospital Barra D’Or, Rede D’Or São Luiz, Rio de Janeiro, RJ, Brasil.; 3 Universidade de São Paulo – USP, Faculdade de Medicina – FM, Hospital das Clínicas – HC, São Paulo, SP, Brasil.; 4 Faculdade de Medicina da Bahia – UFBA, Salvador, BA, Brasil.; 5 Universidade do Estado do Rio de Janeiro – UERJ, Rio de Janeiro, RJ, Brasil.; 6 Universidade Federal do Estado do Rio de Janeiro – UNIRIO, Rio de Janeiro, RJ, Brasil.

**Keywords:** venous thrombosis, disease prevention, pulmonary embolism, patient safety, clinical protocols, risk management

## Abstract

Patients hospitalized for acute medical and surgical illnesses are at risk of developing venous thromboembolism (VTE) during hospitalization and after discharge. Extended pharmacological prophylaxis beyond the hospital stay is recommended for patients undergoing surgeries at high risk for VTE and for selected groups of hospitalized medical patients. This practice involves several challenges, from identification of at-risk populations eligible for extended prophylaxis to choice of the most appropriate anticoagulant and definition of the ideal duration of use. This review will present the main VTE risk assessment models for hospitalized medical and surgical patients, the current recommendations for use of extended prophylaxis, and its limitations and benefits.

## INTRODUCTION

Patients admitted to hospital for acute medical and surgical diseases are at risk of venous thromboembolism (VTE) during and after the hospital stay.^1^ The main risk factors for development of VTE include active cancer, stroke, the prenatal to postnatal cycle, prior VTE, heart failure, trauma, major surgery, obesity, age over 60 years, immobility lasting more than 3 days, inflammatory diseases, sepsis, chronic renal failure, family history of VTE, and hereditary or acquired thrombophilias.^2^^,^ 3

Many healthcare institutions have made efforts to implement hospital protocols for VTE prevention because it is a potentially avoidable complication.^1^ However, the risk of such events is not limited to the period when the patient is in hospital, but extends for up to 3 months after discharge.^1^ Data show that 37% of patients who have VTE in outpatients settings had had a recent hospital admission, 23% had undergone major surgery during the 3 months preceding the event,^4^ and around 67% of cases of VTE occurred during the first month after hospital discharge.^4^ Another important finding is that the incidence of VTE within 100 days of hospital admission is proportional to the number of risk factors that a patient has at discharge: 6.1% in patients with three or more factors and 8.7% in those with four or more.^5^ These data emphasize the opportunity for assessing risk of VTE at the time of discharge for defining the appropriate prophylaxis.

A large American population-based study found that approximately 75% of patients with VTE associated with hospital admissions in the United States are diagnosed around 19.5 days after discharge.^6^ Failure to prevent VTE may be related to insufficient duration of prophylaxis, since the mean time of use of pharmacological prophylaxis found in the study was just 3 days and practically none of the patients were given prophylaxis after discharge.^6^

In the same context, hospitals considered highly effective in terms of VTE prevention did not demonstrate reduced incidence of symptomatic events during the first 90 days after hospitalization.^7^ Institutions with high rates of use of pharmacological prophylaxis during the hospital stay (85.8%) had similar rates of VTE events to institutions with low rates of prophylaxis use (55.5%), at 1.27 and 1.15 events per 10,000 patient-days after hospital discharge, respectively.^7^

These results show the need to use VTE prophylaxis for the correct length of time in high-risk patients and emphasize the impact of this measure on the incidence of events after hospital discharge.^6^^,^ 7 Institutional initiatives to promote awareness of the risk of VTE after discharge could help to guide appropriate use of prophylaxis in vulnerable patients.^1^ Considering human error and the difficulties faced in implementing recommendations for VTE prophylaxis at patient admission, it is important to seek technological strategies that incorporate electronic reminders into medical records at admission and at the time of discharge. One study reported significant increases in rates of pharmacological prophylaxis at hospital discharge when reminders were used (22.0% vs. 9.7%; p < 0.0001), although it did not demonstrate differences in rates of symptomatic VTE at 90 days (4.5% vs. 4.0%; hazard ratio [HR] = 1.12; 95% confidence interval [95%CI] = 0.74–1.69).^8^ The lack of specific recommendations on the type and duration of prophylaxis when the study was conducted may have interfered with correct prescription of long-term thromboprophylaxis after the reminder was sent.

This review will cover models for VTE risk assessment in hospitalized patients and the main strategies for use of extended prophylaxis in medical and surgical patients.

## RECOMMENDED DURATION OF PROPHYLAXIS IN HIGH-RISK MEDICAL AND SURGICAL PATIENTS

The efficacy and safety of thromboprophylaxis with enoxaparin, dalteparin, and fondaparinux in medical patients hospitalized for acute diseases was assessed in the MEDENOX,^9^ PREVENT,^10^ and ARTEMIS^11^ studies respectively. The duration of pharmacological prophylaxis defined as safe and effective for these patients was from 6 to 14 days (mean duration of 7 days).^9^^-^ 11

The ninth version of the American College of Chest Physicians (ACCP) guidelines recommends that hospitalized medical patients at high risk of VTE should be given pharmacological prophylaxis with low molecular weight heparin (LMWH), unfractionated heparin (UFH), or fondaparinux for 6 to 14 days, which can be extended for up to 21 days.^12^ Along similar lines, the National Institute for Health and Care Excellence (NICE) recommends pharmacological prophylaxis for a minimum of 7 days, if the risk of VTE outweighs the risk of bleeding, and recommends LMWH as the first-choice drug.^13^ Extended prophylaxis is defined in the ninth version of the ACCP guidelines as that which is maintained beyond the duration of the standard initial course by 5 to 14 days, for approximately 35 days in total.^12^

Use of direct oral anticoagulants (DOACs) that act to inhibit factor Xa, such as betrixaban (not sold in Brazil until publication of this article) and rivaroxaban, has recently been suggested for extended VTE prophylaxis for up to 45 days in medical patients, after it was approved by the U.S. Food and Drug Administration.^14^ However, given the limitation to subsets of patients who have simultaneously extremely high risk of VTE and low risk of bleeding, the practice has not yet been incorporated into the majority of VTE prophylaxis guidelines, such as the most recent 2018 update by the American Society of Hematology.^15^

In surgical patients, recommendations for extended prophylaxis are better established for the subset of high risk orthopedic patients (varying from 10 to 35 days)^16^ and for major abdominal and pelvic oncological surgeries (4 weeks),^17^^,^ 18 compared with the standard duration of 7 to 10 days, recommended for high risk surgical patients in general.^17^

## VTE AND BLEEDING RISK ASSESSMENT MODELS

Over the last two decades, countless VTE risk assessment models (RAMs) have been released, aiming to organize the most important thromboprophylaxis recommendations in hospitalized patients on the basis of risk stratification.^19^^-^ 25 The RAMs most used globally for estimating risk of VTE in medical patients include the Padua,^19^ IMPROVE (International Medical Prevention Registry on Venous Thromboembolism),^20^ Geneva,^21^ and IMPROVEDD scores (International Medical Prevention Registry on Venous Thromboembolism with D-dimer measurement),^22^ the latter used to estimate the risk of post-discharge VTE. For assessment of risk in surgical patients, the Caprini^23^ and Rogers scores^24^ are recommended, defining the risk of VTE according to the characteristics of the patients and the profile of each surgical procedure. In Brazil, an algorithm based on the Brazilian guidelines for VTE prophylaxis in hospitalized medical patients is frequently employed^25^ as is the algorithm for VTE prevention in surgical patients created by the General Practice Service at the Universidade de São Paulo’s Hospital das Clínicas, based on the seventh ACCP guidelines for VTE prevention and treatment.^26^

The IMPROVE Bleeding Risk Score is the RAM currently validated for assessment of the concomitant risk of bleeding in medical patients.^27^ Patients with scores < 7 can safely be given pharmacological prophylaxis, whereas decisions on prophylaxis should be taken on a case-by-case basis for those with a higher risk of bleeding (scores ≥ 7) who are also at high risk of VTE.^27^

**Figure 1:**
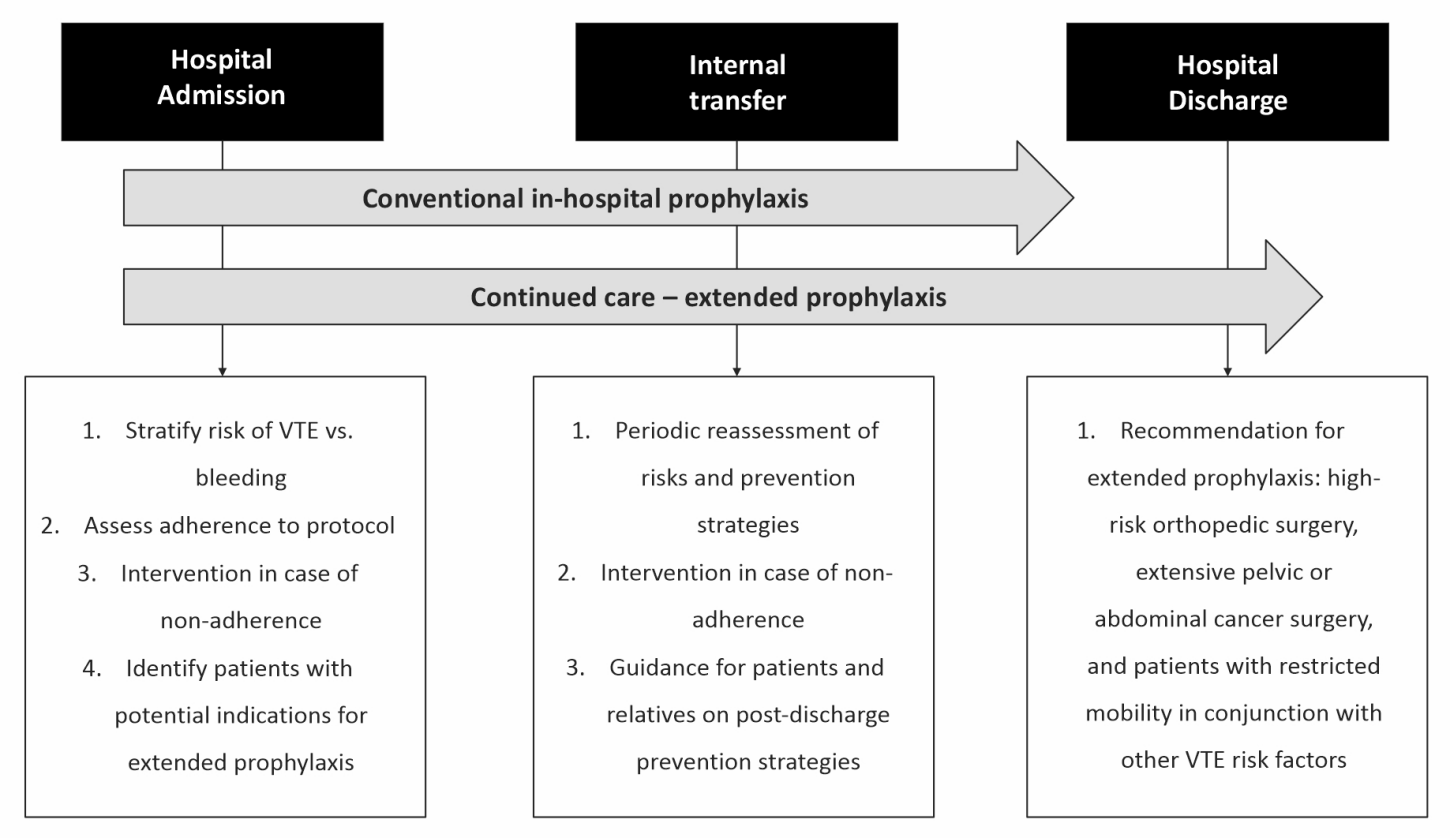
Venous thromboembolism (VTE) prophylaxis care flow.

For patients undergoing surgery, it is necessary to consider the procedure’s potential for risk of bleeding in conjunction with the patient’s individual risk factors in order to define the best strategy for prevention of VTE.^2^ All of the RAMs should be employed systematically and repeatedly at the main stages of care, including hospital admission, transition between sectors, and hospital discharge^2^ (Figure 1). Although these RAMs were designed for stratification of in-hospital VTE risk, they may offer a guide to assessing persistent risk factors and an aid to decision-making on extended pharmacological prophylaxis.

Adaptations have been made to these scores with the objective of identifying potential benefits of extended prophylaxis at discharge. The IMPROVE^20^ score included seven independent VTE risk factors present at admission and while in hospital. D dimer (DD) was added to these factors as a biomarker of additional risk of development of VTE. Patients with an IMPROVEDD score ≥ 2 exhibited greater risk of VTE than those with IMPROVEDD scores of 0 or 1 (HR: 2.73 [95%CI: 1.52–4.90]; p = 0.0007).^22^ IMPROVEDD scores ≥ 2 identified a subset of hospitalized medical patients at increased risk of symptomatic VTE over a 77-day period, contributing to identification of patients who would potentially benefit from extended prophylaxis.^22^ Additionally, patients with IMPROVE scores ≥ 4 or IMPROVE scores of 2 or 3 in conjunction with DD elevation to double the reference values or greater could benefit from extending prophylaxis for up to 45 days after discharge.^28^

## EXTENDED PHARMACOLOGICAL PROPHYLAXIS IN MEDICAL PATIENTS

There is still no consensus on the indications for extended pharmacological prophylaxis in medical patients. Both the ninth ACCP^12^ and the 2018 American Society of Hematology^15^ guidelines suggest that pharmacological prophylaxis should not be extended beyond the duration of immobilization or the acute hospital stay, whereas the International Union of Angiology guidelines^29^ recommend pharmacological prophylaxis post-discharge in female patients, patients older than 75 years or severe immobility, but should be determined on an individual basis.

In the last decade, five large randomized blinded clinical trials assessed the efficacy and safety of extended VTE prophylaxis in hospitalized and acutely ill medical patients^30^^-^ 34 (Table 1). The first of these studies was the EXCLAIM trial (Extended Prophylaxis for Venous Thromboembolism in Acutely Ill Medical Patients with Prolonged Immobilization),^30^ which compared use of enoxaparin to placebo. Studies were later conducted with DOACs: ADOPT (Apixaban Dosing to Optimize Protection from Thrombosis),^31^ MAGELLAN (Multicenter, Randomized, Parallel Group Efficacy and Safety Study for the Prevention of Venous Thromboembolism in Hospitalized Acutely Ill Medical Patients comparing Rivaroxaban with Enoxaparin),^32^ APEX (Acute Medically Ill Venous Prevention with Extended Duration Betrixaban),^33^ and MARINER (Medically Ill Patient Assessment of Rivaroxaban vs. Placebo in Reducing Post-Discharge Venous Thrombo-Embolism Risk).^34^ All of these studies selected large numbers of medical patients who had significantly restricted mobility, defined as total bed rest because of the acute disease for 1 to 3 days or who at most could walk to the bathroom.^30^^-^ 34 In the EXCLAIM,^30^ ADOPT,^31^ APEX,^33^ and MARINER^34^ studies, additional clinical criteria of VTE risk were also employed, such as age ≥ 75 years, prior VTE, or active cancer. In the APEX^33^ and MARINER^34^ studies, the biomarker of risk of VTE of DD ≥ 2 times the upper limit of normality was also used.

**Table 1:** Characteristics of studies of extended prophylaxis in medical patients.

	**EXCLAIM** 30	**ADOPT** 31	**MAGELLAN** 32	**APEX** 33	**MARINER** 34
**Drug**	Enoxaparin 40 mg/day	Apixaban 2.5 mg 2x/day	Rivaroxaban 10 mg/day	Betrixaban 80 mg/day	Rivaroxaban 10 mg/day^*^
**Patients included**	5,963	6,528	8,101	7,513	12,024
**Comparator**	Placebo	Enoxaparin for at least 6 days	Enoxaparin for 10±4 days	Enoxaparin for 10±4 days	Placebo
**Randomization**	In hospital	In hospital	In hospital	In hospital	At hospital discharge
**Risk assessment models**	Not used	Not used	Not used	Not used	IMPROVEDD^22^^,^ 28
**Included D dimer in eligibility criteria**	No	No	No	Yes	Yes
**Duration of treatment**	28±4 days	30 days	35±4 days	35 to 42 days	45 days

* Reduction of rivaroxaban dose to 7.5 mg/day in patients with creatinine clearance > 30 mL/min and < 50 mL/min.

NB: All of the studies listed in this table are multicenter, double-blind, randomized, parallel, clinical intervention trials with evidence level 1B (Oxford Center for Evidence-based Medicine).

ADOPT = *Apixaban Dosing to Optimize Protection from Thrombosis*; APEX = *Acute Medically Ill Venous Prevention with Extended Duration Betrixaban*; EXCLAIM = *Extended Prophylaxis for Venous Thromboembolism in Acutely Ill Medical Patients with Prolonged Immobilization*; MAGELLAN = *Multicenter, Randomized, Parallel Group Efficacy and Safety Study for the Prevention of Venous Thromboembolism in Hospitalized Acutely Ill Medical Patients Comparing Rivaroxaban with Enoxaparin*; MARINER = *Medically Ill Patient Assessment of Rivaroxaban versus Placebo in Reducing Post-Discharge Venous Thromboembolism Risk*.

With relation to the designs of these studies, all of the patients were given pharmacological prophylaxis for the standard minimum period of 6 to 14 days, and were later randomized to continue on the anticoagulant studied or onto placebo for a total period varying from 28 to 45 days.^30^^-^ 34 Patients were randomized to take the medications in analyses conducted from hospital admission onwards, with the exception of the MARINER study,^34^ in which randomization for extended use of rivaroxaban was performed at hospital discharge.

Since risk of bleeding is a factor that limits the overall benefit of extended VTE prophylaxis, the APEX^33^ and MARINER^34^ studies adopted stricter exclusion criteria. They excluded patients with characteristics that could increase the risk of bleeding, as defined by the International Society on Thrombosis and Haemostasis (ISTH),^34^ such as presence of bronchiectasis, pulmonary cavitations, active cancer, active gastroduodenal ulcer, history of bleeding in the previous 3 months, and antiplatelet treatment.

The primary efficacy objectives were similar in these clinical trials. The EXCLAIM,^30^ ADOPT,^31^ MAGELLAN,^32^ and APEX^33^ studies evaluated incidence of deep venous thrombosis (DVT), symptomatic or not, associated with nonfatal pulmonary thromboembolism (PTE) and death from VTE, whereas the MARINER study^34^ did not include asymptomatic events in its analysis. With regard to safety-related outcomes, the primary safety outcome in the EXCLAIM,^30^ APEX,^33^ and MARINER studies was rates of major bleeding, defined as a ≥ 2 g fall in hemoglobin, transfusion of ≥ 2 units of packed red blood cells, bleeding in a critical organ, or fatal bleeding.^34^ The ADOPT^31^ and MAGELLAN^32^ studies assessed a combination of major bleeding and non-major clinically relevant (NMCR) bleeding, defined as general bleeding that did not fit the criteria for major bleeding, but was associated with a need for unplanned medical intervention, temporary suspension of treatment, or patient discomfort such as pain or compromised daily activities.^32^

The EXCLAIM study^30^ assessed the risk of VTE associated with immobility defined at two levels (level 1: absolute bed rest; and level 2: bathroom privileges) in medical patients with acute diseases on enoxaparin for a period of 28 ± 4 days. There was a reduction in VTE rate from 4.0% to 2.5% (p < 0.003), but the benefit of extending pharmacological prophylaxis was limited to female patients over the age of 75 years who were in absolute bed rest. The rate of major bleeding was higher on enoxaparin than with placebo (0.8% vs. 0.3%; p = 0.02).^30^

In the ADOPT study,^31^ a prolonged course of pharmacological prophylaxis with apixaban in acutely ill medical patients was not superior to a shorter course of enoxaparin and was also associated with higher major bleeding rates than LMWH (0.47% vs. 0.19%; p = 0.04).

The primary composite efficacy objective of the MAGELLAN study^32^ was defined as noninferiority on the 10th day of the study and superiority on the 35th day. The primary safety endpoint was major bleeding or NMCR bleeding. The noninferiority efficacy result was observed on the 10th day (2.7% in both groups; p = 0.003). There was a 23% reduction in events related to the primary objective on the 35th day with rivaroxaban, compared to placebo (4.4% vs. 5.7%; p = 0.02). However, there were increases in major bleeding (1.1% vs. 0.4%; p < 0.001) and in NMCR bleeding (4.1% vs. 1.7%; p < 0.001) on the 35th day. It should be pointed out that this study included patients at high risk of VTE and also with a high risk of bleeding, resulting in a reduction in VTE events, but at the expense of higher bleeding rates.

In the APEX study,^33^ acutely ill medical patients with DD levels ≥ 2 times the upper limit of normality were randomized into three different betrixaban cohorts. The prespecified composite primary objective was asymptomatic proximal DVT, proximal symptomatic DVT, and fatal or nonfatal PTE, which was not achieved, with a statistical value that was borderline (p = 0.054). However, the results for the secondary preestablished objectives were achieved. Extended prophylaxis with betrixaban reduced the risk of symptomatic VTE and hospital readmission and the incidence of stroke and cardiovascular events compared with standard prophylaxis with enoxaparin.^1^^,^ 33 In contrast with what was observed in other clinical trials,^30^^-^ 34 betrixaban was not associated with major bleeding in the comparison of the three cohorts, but there was approximately twice the rate of NMCR bleeding.^33^ Based on these results, in 2017 the Food and Drug Administration approved betrixaban for extended VTE prophylaxis in patients with acute medical diseases;^1^^,^ 35 but it was not approved by the European Medicine Agency^35^ This was the first study to establish similar efficacy for reduction of VTE rates between a DOAC and enoxaparin in medical patients put on extended prophylaxis, without causing increased rates of major bleeding.^33^

Next, the MARINER study^34^ evaluated the efficacy and safety of rivaroxaban 10 mg/day, started at hospital discharge and maintained for 45 days, compared with placebo.^34^^,^ 36 Patients were selected using the IMPROVEDD score.^22^^,^ 28 Rivaroxaban, in prophylactic doses, administered after hospital discharge did not achieve the composite primary objective of reducing symptomatic VTE and death related to VTE.^35^ However, there were significant reductions in nonfatal symptomatic VTE (HR: 0.44; 95%CI: 0.22–0.89) and all causes mortality (HR: 0.73; 95%CI: 0.54–0.97; p = 0.033), without increased risk of major bleeding. A subanalysis of the MAGELLAN study^32^ was conducted attempting to identify the population that would most benefit from extended prophylaxis, i.e., those with high risk of VTE but low risk of bleeding, applying the exclusion criteria described above for the MARINER study^34^ (MARINER-like MAGELLAN subset).^37^ In this subanalysis, the benefits of extended prophylaxis were maintained and there was no increase in the rates of major bleeding compared with placebo, although the rates of NMCR bleeding were higher, enabling selection of a patient profile with potential benefit from extended prophylaxis.^37^ Thus, in 2019, rivaroxaban was approved for use in hospital and extended prophylaxis in medical patients in the United States.^1^^,^ 35

A meta-analysis of five studies of extended pharmacological prophylaxis in medical patients was published recently.^38^ Around 40,000 patients were analyzed (mean age ranged from 67 to 77 years, 48 to 54% of patients were female, and congestive heart failure was the main cause of hospital admission), demonstrating a reduction in symptomatic VTE events or VTE-related deaths compared with standard prophylaxis (0.8% vs. 1.2%; relative risk [RR]: 0.61; 95%CI: 0.44–0.83; p = 0.002). However, there was an increased risk of major or fatal hemorrhage (0.6% vs. 0.3%; RR: 2.04; 95%CI: 1.42–2.91; p < 0.001). The analysis demonstrated that the number needed to treat (NNT) to prevent one symptomatic VTE event or VTE-related death was 250, while the number needed to harm (NNH) to cause a primary or fatal hemorrhagic event was 333.^38^ The analysis acknowledges the several limitations of the studies assessed, such as the variations in inclusion criteria and duration of extended pharmacological prophylaxis, beyond the protocols used to diagnose VTE.^38^ The results of this meta-analysis present an important reflection of the need to balance the decision between the efficacy and desirable benefits of extended prophylaxis against the safety and potential damage of using it, since the NNT to avoid a symptomatic event (250) was very close to the NNH (333) to cause major bleeding.^38^

## EXTENDED PHARMACOLOGICAL PROPHYLAXIS IN SURGICAL PATIENTS

Recommendations for assessing the duration of pharmacological VTE prophylaxis in surgical patients have been proposed based on the Caprini score,^23^^,^ 39 ^,^ 40 originally validated for general, pelvic, vascular, bariatric, and reconstructive plastic surgery. For patients classified as at moderate risk (Caprini score of 3 or 4), only in-hospital pharmacological prophylaxis is recommended. Pharmacological prophylaxis is recommended for 7 to 10 days in high-risk patients (Caprini scores 5 to 8). In patients at very high risk (Caprini scores > 8), prolonged prophylaxis should be prescribed for 30 days, unless there are contraindications^39^^,^ 40 (Table 2). The very high risk group includes patients undergoing major orthopedic surgery, such as elective total hip replacement (THR) or total knee replacement (TKR), patients undergoing surgery for fractured pelvis or hips, and patients being treated for severe trauma or spine injuries, and those undergoing extensive abdominal or pelvic cancer surgery.^16^^,^ 17

**Table 2:** Recommendations for venous thromboembolism prophylaxis regimes and duration in surgical patients, based on the Caprini score.

**Caprini score**	**Risk categories**	**Prophylaxis recommendation**	**Duration of prophylaxis**
0	Very low	Early and frequent mobilization only or according to the surgical team’s assessment: IPC or low dose of UFH or LMWH	During hospital stay
1-2	Low	IPC or low dose of UFH or LMWH	During hospital stay
3-4	Moderate	IPC and low dose of UFH or LMWH	During hospital stay
5-8	High	IPC and low dose of UFH or LMWH	7 to 10 days in total
> 8	Very high	IPC and low dose of UFH or LMWH	30 days in total

UFH = unfractionated heparin; LMWH = low molecular weight heparin; IPC = intermittent pneumatic compression.

Adapted from: Cassidy et al.^39^

All patients with malignant cancer undergoing major surgical interventions should be given pharmacological prophylaxis with UFH or LMWH, unless contraindicated because of active bleeding or high potential risk of bleeding.^17^^,^ 18 Extended prophylaxis with LMWH for up to 4 weeks during the postoperative period is recommended for patients who undergo major open or laparoscopic abdominal or pelvic surgery for cancer.^17^ The most recent American Society of Clinical Oncology guidelines recommend LMWH for up to 4 weeks during the postoperative period after major abdominal or pelvic surgery, in cancer patients who have the characteristics of high risk of VTE: restricted mobility, obesity, prior VTE, or additional risk factors. In lower risk situations the decision should be individualized.^18^

Other groups of surgical patients for whom extended prophylaxis is well-established include those undergoing elective TKR or THR and those having surgery to repair proximal femur fractures (PFF).^16^^,^ 41 Pharmacological prophylaxis is recommended for a minimum of 10 to 14 days for TKR and 28 to 35 days for THR with LMWH, UFH, fondaparinux, apixaban, dabigatran, rivaroxaban, warfarin, or acetylsalicylic acid (ASA), or mechanical prophylaxis with intermittent pneumatic compression (IPC) for those at high risk of bleeding.^16^ For patients undergoing TKR, the ninth ACCP guidelines recommend extending prophylaxis for up to 35 days after surgery, compared with use limited to 10 to 14 days. In patients who undergo PFF repair, the duration of prophylaxis should be from 28 to 35 days and UFH, LMWH, or fondaparinux should be used, since DOACs have not been approved for this purpose.^16^^,^ 41 For patients undergoing THR, TKR, or PFF surgery, combined use of pharmacological prophylaxis and mechanical prophylaxis with IPC of the lower limbs is recommended during the hospital stay.^16^

## STRATEGIES TO OPTIMIZE ADHERENCE TO VTE PROPHYLAXIS AT HOSPITAL DISCHARGE

Although the risk of VTE after hospital discharge is widely recognized among high-risk patients, studies demonstrate that extended prophylaxis is still underutilized.^42^^,^ 43 Healthcare institutions should focus their efforts on identification of barriers that limit adhesion to this safety practice and on implementation of facilitating strategies.

Considering that the length of hospital stay is often shorter than the total recommended duration of anticoagulation, thromboprophylaxis guidelines should be adapted to fit early hospital discharge strategies, avoiding events after discharge and readmission for VTE.^1^ Therefore, planning of hospital discharge is an essential element in ensuring care transition and the overall efficacy of thromboprophylaxis in different clinical settings.^1^^,^ 41 Several strategies can be incorporated into care routines to maintain the quality and safety of VTE prevention (Table 3).

**Table 3:** Actions to facilitate implementation of safe discharge for prevention of venous thromboembolism (VTE).

Institutional VTE prophylaxis protocol including extended prophylaxis recommendations
Routine use of VTE RAMs
Technological strategies incorporating electronic reminders in patient records at admission and hospital discharge
Treatment plans that incorporate the main stages of institutional care
Active screening for patients eligible for extended pharmacological prophylaxis at admission
Use of a discharge planning checklist
Development of multidisciplinary discharge summaries
Training a multidisciplinary team to provide discharge guidance
Development of educational materials for patients and relatives
Creation of hospital discharge commissions

RAMs = risk assessment models.

Adapted from: Barkoudah et al.^1^

## CONCLUSIONS

The elevated incidence of VTE events after hospital discharge underscores the need for individualized VTE risk assessment in medical and surgical patients at discharge. Reduction of the rate of late events is dependent on identification of groups of patients who can potentially benefit from pharmacological prophylaxis for longer periods, without increased risk of bleeding.^38^ These definitions are clearer for high risk surgical orthopedic and cancer patients. In medical patients, extending prophylaxis confers benefits in terms of prevention of events,^30^^-^ 34 but increases rates of major bleeding^30^^-^ 32 and NMCR bleeding,^32^^-^ 34 even in populations that meet more rigid criteria applied to exclude patients with greater hemorrhagic risk. Use of multiple strategies to increase adherence to thromboprophylaxis protocols and measurement of results with proposals for improvements are the most important institutional actions to guarantee adequate protection of patients.
