# Supplementary material for: Challenges of extended venous thromboembolism prophylaxis in medical and surgical patients
Source: J Vasc Bras. 2022 Jun 27;21:e20210195. doi: 10.1590/1677-5449.202101951 (PMC9250351; doi:10.1590/1677-5449.202101951)
Supplement: Translation

# Translation: Desafios da profilaxia estendida do tromboembolismo venoso em pacientes clínicos e cirúrgicos

**DOI:** 10.1590/1677-5449.202101952

**Authors:** Maria Chiara Chindamo, Edison Ferreira Paiva, Plinio Resende do Carmo, Ana Thereza Cavalcanti Rocha, Marcos Arêas Marques

**Affiliations:** 1 Universidade Federal do Rio de Janeiro – UFRJ, Rio de Janeiro, RJ, Brasil.; 2 Hospital Barra D’Or, Rede D’Or São Luiz, Rio de Janeiro, RJ, Brasil.; 3 Universidade de São Paulo – USP, Faculdade de Medicina – FM, Hospital das Clínicas – HC, São Paulo, SP, Brasil.; 4 Faculdade de Medicina da Bahia – UFBA, Salvador, BA, Brasil.; 5 Universidade do Estado do Rio de Janeiro – UERJ, Rio de Janeiro, RJ, Brasil.; 6 Universidade Federal do Estado do Rio de Janeiro – UNIRIO, Rio de Janeiro, RJ, Brasil.

**Keywords:** trombose venosa profunda, prevenção de doenças, embolia pulmonar, segurança do paciente, protocolos clínicos, gestão do risco

## Abstract

Pacientes hospitalizados por doenças clínicas e cirúrgicas agudas estão sob risco de desenvolvimento de tromboembolismo venoso (TEV) durante a hospitalização e após a alta. A profilaxia farmacológica estendida além do período da hospitalização é recomendada para pacientes submetidos a cirurgias de alto risco de TEV e para grupos selecionados de pacientes clínicos hospitalizados. Diversos desafios envolvem essa prática, desde o reconhecimento das populações de risco elegíveis para a extensão da profilaxia até a escolha do anticoagulante mais adequado e a definição do tempo ideal de utilização. Os principais modelos de avaliação de risco de TEV em pacientes clínicos e cirúrgicos hospitalizados, as recomendações atuais para uso da profilaxia estendida e suas limitações e benefícios serão apresentados nesta revisão.

## INTRODUÇÃO

Pacientes hospitalizados por doenças clínicas e cirúrgicas agudas apresentam risco de tromboembolismo venoso (TEV) durante e após a internação hospitalar^1^. Os fatores de risco para o desenvolvimento de TEV incluem principalmente a presença de câncer ativo, acidente vascular cerebral, ciclo gravídico puerperal, TEV prévio, insuficiência cardíaca, traumas, cirurgias maiores, obesidade, idade maior que 60 anos, imobilidade superior a 3 dias, doenças inflamatórias, sepse, insuficiência renal crônica, história familiar de TEV e trombofilias hereditárias ou adquiridas^2^^,^ 3.

Esforços na implementação de protocolos de prevenção do TEV hospitalar têm sido o foco de muitas instituições de saúde por tratar-se de uma complicação potencialmente evitável^1^. O risco de eventos, no entanto, não está limitado apenas ao período da hospitalização, estendendo-se por até 3 meses após a alta^1^. Dados apontam que 37% dos pacientes que apresentaram TEV ambulatorialmente tiveram hospitalização recente, 23% foram submetidos a cirurgias de grande porte nos 3 meses que antecederam o evento^4^ e em torno de 67% dos casos de TEV ocorreram no primeiro mês após a alta hospitalar^4^. Outro aspecto importante é o fato de que a incidência de TEV em até 100 dias após a hospitalização é proporcional ao número de fatores de risco que o paciente apresenta na alta: 6,1% naqueles com três ou mais fatores e 8,7% naqueles com quatro ou mais^5^. Esses dados reforçam a oportunidade de avaliação do risco de TEV no momento da desospitalização para definição da profilaxia adequada.

Um grande estudo populacional americano documentou que aproximadamente 75% dos pacientes com TEV associado à hospitalização nos Estados Unidos são diagnosticados cerca de 19,5 dias após a alta^6^. A falha na prevenção do TEV pode estar relacionada à duração inadequada da profilaxia, uma vez que o tempo médio de utilização de farmacoprofilaxia avaliada no estudo foi de apenas 3 dias e praticamente nenhum paciente recebeu profilaxia após a alta^6^.

Ainda nesse contexto, hospitais considerados de alta eficiência com relação à prevenção de TEV não demonstraram queda na incidência de eventos sintomáticos no período de 90 dias após a hospitalização^7^. Taxas semelhantes de eventos de TEV de 1,27 e 1,15 por 10.000 pacientes-dia após a alta hospitalar foram encontradas, respectivamente, em instituições com adesão alta do uso de farmacoprofilaxia durante a internação (85,8%) comparadas a instituições com baixa aderência ao seu uso (55,5%)^7^.

Esses resultados apontam a necessidade do uso da profilaxia de TEV pelo tempo correto em pacientes de alto risco e enfatizam o impacto dessa medida na incidência de eventos após a alta hospitalar^6^^,^ 7. Iniciativas institucionais para promover a conscientização sobre o risco de TEV após a alta podem ajudar a direcionar o uso apropriado da profilaxia em pacientes vulneráveis^1^. Dadas as falhas humanas e dificuldades de implementar recomendações de profilaxia de TEV na admissão de pacientes, é importante buscar estratégias de tecnologia que incorporem alertas eletrônicos aos prontuários médicos na admissão e no momento da desospitalização. Um estudo revelou significativo aumento das taxas de farmacoprofilaxia na alta hospitalar com o uso de alertas (22,0% vs. 9,7%; p < 0,0001), embora não tenha demonstrado diferenças nas taxas de TEV sintomático em 90 dias (4,5% vs. 4,0%; *hazard ratio* [HR] = 1,12; intervalo de confiança de 95% [IC95%] = 0,74–1,69)^8^. A falta de recomendações específicas na ocasião sobre o tipo e a duração da profilaxia poderia ter dificultado a prescrição adequada de tromboprofilaxia de longa duração após o alerta.

Nesta revisão, serão abordados os modelos de avaliação de risco de TEV em pacientes hospitalizados e as principais estratégias de uso da profilaxia estendida em pacientes clínicos e cirúrgicos.

## TEMPO RECOMENDADO DE PROFILAXIA EM PACIENTES CLÍNICOS E CIRÚRGICOS DE ALTO RISCO

A eficácia e a segurança da tromboprofilaxia com enoxaparina, dalteparina e fondaparinux em pacientes clínicos hospitalizados por doença aguda foram avaliadas nos estudos MEDENOX^9^, PREVENT^10^ e ARTEMIS^11^, respectivamente. O período de utilização de farmacoprofilaxia definido como eficaz e seguro para esses pacientes foi de 6 a 14 dias (média de 7 dias)^9^^-^ 11.

A nona versão da diretriz do *American College of Chest Physicians* (ACCP) recomenda que os pacientes clínicos hospitalizados com alto risco de TEV recebam farmacoprofilaxia com heparina de baixo peso molecular (HBPM), heparina não fracionada (HNF) ou fondaparinux por 6 a 14 dias, podendo ser estendida por até 21 dias^12^. Da mesma forma, o *National Institute for Health and Care Excellence* (NICE) indica a farmacoprofilaxia pelo período mínimo de 7 dias, se o risco de TEV ultrapassar o risco de sangramento, e recomenda a HBPM como o medicamento de primeira escolha^13^. A profilaxia estendida é definida na nona versão da diretriz do ACCP como aquela mantida após o curso inicial padrão de 5 a 14 dias por até aproximadamente 35 dias, no total^12^.

O uso de anticoagulantes orais de ação direta (DOACs) que atuam na inibição do fator Xa, como betrixabana (não comercializada no Brasil até a publicação deste artigo) e rivaroxabana, vem sendo recentemente pleiteado para a profilaxia estendida de TEV por até 45 dias em pacientes clínicos, após aprovação pela agência regulatória americana^14^. No entanto, dadas as limitações dos subgrupos de pacientes que apresentam concomitantemente altíssimo risco de TEV e baixo risco de sangramento, essa prática ainda não foi incorporada pela maioria das diretrizes de profilaxia de TEV, como a última atualização de 2018 da *American Society of Hematology* 15.

Nos pacientes cirúrgicos, as recomendações de profilaxia estendida estão melhor estabelecidas para o grupo de pacientes ortopédicos de alto risco (variando de 10 a 35 dias)^16^ e nas cirurgias oncológicas abdominais e pélvicas maiores (4 semanas)^17^^,^ 18 em comparação ao período padrão de 7 a 10 dias recomendado para pacientes cirúrgicos de alto risco em geral^17^.

## MODELOS DE AVALIAÇÃO DO RISCO DE TEV E SANGRAMENTO

Ao longo das últimas duas décadas, inúmeros modelos de avaliação de risco (MARs) de TEV foram disponibilizados com o objetivo de direcionar as principais recomendações de tromboprofilaxia hospitalar com base na estratificação de risco^19^^-^ 25. Entre os mais utilizados em pacientes clínicos em todo o mundo, estão os escores de Padua^19^, IMPROVE (*International Medical Prevention Registry on Venous Thromboembolism*)^20^, de Genebra^21^ e IMPROVEDD (*International Medical Prevention Registry on Venous Thromboembolism with D-dimer measurement*), este último utilizado para estimativa do risco de TEV pós-alta^22^. Para avaliação de risco em pacientes cirúrgicos, são recomendados o escore de Caprini^23^ e o de Rogers^24^, que definem o risco de TEV de acordo com as características dos pacientes e do perfil de cada cirurgia. No Brasil, são frequentemente empregados o algoritmo da diretriz brasileira de profilaxia de TEV em pacientes clínicos hospitalizados^25^ e o algoritmo de prevenção de TEV em pacientes cirúrgicos, criado no Serviço de Clínica Geral do Hospital das Clínicas da Faculdade de Medicina da Universidade de São Paulo e baseado na sétima diretriz do ACCP de prevenção e tratamento do TEV^26^.

Para avaliação concomitante do risco de sangramento de pacientes clínicos, o MAR validado até o momento é o IMPROVE *Bleeding Risk Score* 27. Os pacientes com pontuação < 7 podem receber farmacoprofilaxia com segurança, enquanto as decisões relativas à profilaxia devem ser tomadas de forma individualizada para aqueles com alto risco de sangramento (pontuação ≥ 7) e alto risco de TEV^27^.

Nos pacientes submetidos à cirurgia, é necessário considerar o potencial risco de sangramento do procedimento em conjunto com os fatores de risco individuais para definir a melhor estratégia de prevenção de TEV^2^. Todos os MARs devem ser utilizados de forma sistemática e repetida nas principais etapas da assistência, incluindo admissão hospitalar, transição entre setores e desospitalização^2^ (Figura 1). Apesar de esses MARs terem sido desenhados para estratificação do risco de TEV durante a internação, podem representar um norteador para avaliação da persistência de fatores de risco e auxiliar na tomada de decisão sobre a farmacoprofilaxia estendida.

**Figura 1:** Fluxo de assistência na profilaxia de tromboembolismo venoso (TEV).

Adaptações de escores foram realizadas com o objetivo de identificar potenciais benefícios da profilaxia estendida na alta. Ao escore IMPROVE^20^, que inclui sete fatores de risco independentes de TEV presentes na admissão e durante a internação, foi adicionado o dímero D (DD) como um biomarcador de risco adicional para o desenvolvimento de TEV. Pacientes com uma pontuação de IMPROVEDD ≥ 2 apresentaram um risco maior de TEV em comparação àqueles com valores de IMPROVEDD de 0 a 1 (HR: 2,73 [IC95%: 1,52–4,90]; p = 0,0007)^22^. A pontuação do IMPROVEDD ≥ 2 identificou um subgrupo de pacientes clínicos hospitalizados com risco aumentado de TEV sintomático ao longo de 77 dias, contribuindo para a identificação de pacientes com potencial benefício da profilaxia estendida^22^. Adicionalmente, pacientes com escore IMPROVE ≥ 4 ou escore IMPROVE 2 ou 3 associado a elevação de duas ou mais vezes dos valores de referência do DD poderiam beneficiar-se da extensão da profilaxia por até 45 dias após a alta^28^.

## FARMACOPROFILAXIA ESTENDIDA EM PACIENTES CLÍNICOS

Ainda não há um consenso quanto à indicação de farmacoprofilaxia estendida em pacientes clínicos. Tanto a nona ACCP^12^ quanto a diretriz da *American Society of Hematology* publicada em 2018^15^ sugerem contra a extensão de farmacoprofilaxia além do período de imobilização ou do período agudo de hospitalização, enquanto a diretriz da *International Union of Angiology* 29 considera a farmacoprofilaxia pós-alta em mulheres, em pacientes com mais de 75 anos de idade ou em pacientes com imobilidade significativa, mas deve ser determinada individualmente.

Na última década, cinco grandes ensaios clínicos cegos e randomizados avaliaram a eficácia e a segurança da profilaxia estendida de TEV em pacientes clínicos hospitalizados e agudamente doentes^30^^-^ 34 (Tabela 1). O primeiro estudo foi o EXCLAIM (*Extended Prophylaxis for Venous Thromboembolism in Acutely Ill Medical Patients with Prolonged Immobilization*)^30^, que comparou o uso de enoxaparina ao placebo. Na sequência, foram realizados estudos com DOACs: ADOPT (*Apixaban Dosing to Optimize Protection from Thrombosis*)^31^, MAGELLAN (*Multicenter, Randomized, Parallel Group Efficacy and Safety Study for the Prevention of Venous Thromboembolism in Hospitalized Acutely Ill Medical Patients Comparing Rivaroxaban with Enoxaparin*)^32^, APEX (*Acute Medically Ill Venous Prevention with Extended Duration Betrixaban*)^33^ e MARINER (*Medically Ill Patient Assessment of Rivaroxaban versus Placebo in Reducing Post-Discharge Venous Thrombo-Embolism Risk*)^34^. Todos esses estudos selecionaram um número expressivo de pacientes clínicos que apresentavam perda significativa de mobilidade definida como restrição total ao leito decorrente de doença aguda por período de 1 a 3 dias ou que, no máximo, conseguiam locomover-se até o banheiro^30^^-^ 34. Nos estudos EXCLAIM^30^, ADOPT^31^, APEX^33^ e MARINER^34^, critérios clínicos adicionais de risco de TEV, como idade ≥ 75 anos, TEV prévio e câncer em atividade, também foram empregados. O biomarcador de risco de TEV, representado pelo DD ≥ 2 vezes o limite superior de normalidade, também foi utilizado nos estudos APEX^33^ e MARINER^34^.

**Tabela 1:** Características dos estudos de profilaxia estendida em pacientes clínicos.

	**EXCLAIM** 30	**ADOPT** 31	**MAGELLAN** 32	**APEX** 33	**MARINER** 34
**Droga**	Enoxaparina 40 mg/dia	Apixabana 2,5 mg 2x/dia	Rivaroxabana 10 mg/dia	Betrixabana 80 mg/dia	Rivaroxabana 10 mg/dia*
**Pacientes incluídos**	5.963	6.528	8.101	7.513	12.024
**Comparador**	Placebo	Enoxaparina por pelo menos 6 dias	Enoxaparina por 10±4 dias	Enoxaparina por 10±4 dias	Placebo
**Randomização**	No hospital	No hospital	No hospital	No hospital	Na alta hospitalar
**Modelos de avaliação de risco**	Não utilizado	Não utilizado	Não utilizado	Não utilizado	IMPROVEDD^22^^,^ 28
**Inclusão do dímero D para elegibilidade**	Não	Não	Não	Sim	Sim
**Duração do tratamento**	28±4 dias	30 dias	35±4 dias	35 a 42 dias	45 dias

* Redução da dose de rivaroxabana para 7,5 mg/dia em pacientes com *clearance* de creatinina > 30 mL/min e < 50 mL/min.

Obs.: Todos estudos referenciados nesta tabela são ensaios clínicos intervencionistas, randomizados, duplo-cegos, paralelos e multicêntricos, com nível de evidência 1B (*Oxford Centre for Evidence-based Medicine*).

ADOPT = *Apixaban Dosing to Optimize Protection from Thrombosis*; APEX = *Acute Medically Ill Venous Prevention with Extended Duration Betrixaban*; EXCLAIM = *Extended Prophylaxis for Venous Thromboembolism in Acutely Ill Medical Patients with Prolonged Immobilization*; MAGELLAN = *Multicenter, Randomized, Parallel Group Efficacy and Safety Study for the Prevention of Venous Thromboembolism in Hospitalized Acutely Ill Medical Patients Comparing Rivaroxaban with Enoxaparin*; MARINER = *Medically Ill Patient Assessment of Rivaroxaban versus Placebo in Reducing Post-Discharge Venous Thromboembolism Risk*.

Com relação ao desenho dos estudos, todos os pacientes receberam farmacoprofilaxia pelo período mínimo padrão de 6 a 14 dias, tendo sido posteriormente randomizados para continuar com o anticoagulante estudado ou com placebo por um período total que variou de 28 a 45 dias^30^^-^ 34. Os pacientes foram randomizados para utilização do medicamento em análise a partir da internação hospitalar, com exceção do estudo MARINER^34^, cuja randomização para o uso estendido de rivaroxabana ocorreu a partir da alta hospitalar.

Como o risco de sangramento é um fator limitante quanto ao benefício global da profilaxia estendida de TEV, os estudos APEX^33^ e MARINER^34^ adotaram critérios mais rígidos de exclusão. Foram excluídos dos estudos pacientes com características que pudessem aumentar o risco de sangramento, conforme definido pela International Society on Thrombosis and Haemostasis (ISTH)^34^, como a presença de bronquiectasias, cavitações pulmonares, câncer ativo, úlcera gastroduodenal ativa, história de sangramento nos últimos 3 meses e uso de terapia antiplaquetária.

Os objetivos primários de eficácia dos ensaios clínicos foram semelhantes. Os estudos EXCLAIM^30^, ADOPT^31^, MAGELLAN^32^ e APEX^33^ avaliaram a incidência de trombose venosa profunda (TVP), sintomática ou não, associada a tromboembolismo pulmonar (TEP) não fatal e a morte por TEV, enquanto o estudo MARINER^34^ não incluiu a avaliação de eventos assintomáticos. Com relação aos desfechos de segurança, as taxas de sangramento maior, definidas como queda ≥ 2 g de hemoglobina, transfusão ≥ 2 concentrados de hemácias e sangramento em órgão crítico ou fatal, foram o objetivo primário de segurança dos estudos EXCLAIM^30^, APEX^33^ e MARINER^34^. Os estudos ADOPT^31^ e MAGELLAN^32^ avaliaram a combinação de sangramento maior com sangramento não maior clinicamente relevante (NMCR). Este último foi definido como sangramento geral que não atende aos critérios de sangramento maior, mas está associado à necessidade de intervenção médica não programada, cessação temporária do tratamento ou desconforto para o paciente, como dor ou prejuízo das atividades de vida diária^32^.

No estudo EXCLAIM^30^, foi avaliado o risco de TEV associado à imobilidade definida em dois níveis (nível 1: repouso absoluto no leito; e nível 2: privilégio de ir ao banheiro) em pacientes clínicos com doenças agudas sob uso de enoxaparina por um período de 28 ± 4 dias. Houve redução das taxas de TEV de 4,0% para 2,5% (p < 0,003); no entanto, o benefício da extensão da farmacoprofilaxia foi limitado a pacientes do sexo feminino com idade superior a 75 anos que se encontravam em repouso absoluto no leito. A taxa de sangramento maior foi superior com o uso de enoxaparina em comparação com placebo (0,8% vs. 0,3%; p = 0,02)^30^.

No estudo ADOPT^31^, um curso prolongado de farmacoprofilaxia com apixabana em pacientes clínicos agudamente enfermos não foi superior a um curso mais curto de enoxaparina, além de apresentar taxas de sangramento maior superiores às da HBPM (0,47% vs. 0,19%; p = 0,04).

No estudo MAGELLAN^32^, o objetivo primário composto de eficácia foi definido como de não inferioridade no 10º dia do estudo e de superioridade no 35º dia. O objetivo primário de segurança foi sangramento maior ou sangramento NMCR. O resultado de não inferioridade para eficácia foi encontrado no 10º dia (2,7% em ambos os grupos; p = 0,003). Houve redução de 23% dos eventos relacionados ao objetivo primário no 35º dia com uso da rivaroxabana, em comparação com placebo (4,4% vs. 5,7%; p = 0,02). No entanto, houve aumento de sangramento maior (1,1% vs. 0,4%; p < 0,001) e de sangramento NMCR (4,1% vs. 1,7%; p < 0,001) no 35º dia. Cabe ressaltar que esse estudo incluiu pacientes de alto risco de TEV e também de alto risco de sangramento, resultando em redução dos eventos de TEV, porém às custas de maiores taxas de sangramento.

No estudo APEX^33^, os pacientes clínicos agudamente doentes e com valores de DD ≥ 2 vezes o limite superior de normalidade foram randomizados em três coortes distintas para o uso da betrixabana. O objetivo primário composto de eficácia pré-especificada, definido como TVP proximal assintomática, TVP proximal sintomática, TEP fatal ou não fatal, não foi alcançado, apresentando valor estatístico limítrofe (p = 0,054). No entanto, os resultados dos objetivos secundários pré-especificados foram obtidos. A profilaxia estendida com betrixabana reduziu o risco de TEV sintomático, a readmissão hospitalar e a incidência de acidente vascular encefálico e de eventos cardiovasculares em comparação com a profilaxia padrão com enoxaparina^1^^,^ 33. Ao contrário do observado em outros ensaios clínicos^30^^-^ 34, o uso de betrixabana não foi associado a sangramento maior na comparação das três coortes, mas houve frequência aproximadamente duas vezes maior de sangramento NMCR^33^. Com base nesses resultados, a *Food and Drug Administration* aprovou a betrixabana em 2017 para profilaxia estendida de TEV em pacientes com doenças clínicas agudas^1^^,^ 35; no entanto, não houve aprovação pela agência regulatória europeia (*European Medicine Agency*)^35^. Esse foi o primeiro estudo a estabelecer semelhança de eficácia na redução de taxas de TEV entre um DOAC e a enoxaparina em pacientes clínicos submetidos a profilaxia estendida, sem causar aumento das taxas de sangramento maior^33^.

Em sequência, o estudo MARINER^34^ avaliou a eficácia e a segurança da rivaroxabana 10 mg/dia, iniciada na alta hospitalar e continuada por 45 dias, em comparação com placebo^34^^,^ 36. Os pacientes foram selecionados a partir da utilização do escore IMPROVEDD^22^^,^ 28. A rivaroxabana, em dose profilática, administrada após a alta hospitalar não atingiu o objetivo primário composto de redução de TEV sintomático e morte relacionada ao TEV^35^. No entanto, houve redução significativa de TEV sintomático não fatal (HR: 0,44; IC95%: 0,22–0,89) e mortalidade por todas as causas (HR: 0,73; IC95%: 0,54–0,97; p = 0,033), sem aumento do risco de sangramento maior. Buscando identificar a população com maior benefício da profilaxia estendida, ou seja, com alto risco de TEV e baixo risco de sangramento, foi realizada uma subanálise do estudo MAGELLAN^32^ aplicando-se os critérios de exclusão do estudo MARINER^34^ acima descritos (subgrupo MAGELLAN MARINER-*like*)^37^. Nessa subanálise, os benefícios da profilaxia estendida foram mantidos, e não houve aumento das taxas de sangramento maior em comparação com placebo, embora as taxas de sangramento NMCR ainda tivessem sido superiores, permitindo selecionar um perfil de pacientes com benefício potencial para a profilaxia estendida^37^. Dessa forma, a rivaroxabana recebeu aprovação para uso na profilaxia hospitalar e estendida em pacientes clínicos nos Estados Unidos a partir de 2019^1^^,^ 35.

Uma metanálise dos cinco estudos de farmacoprofilaxia estendida em pacientes clínicos foi publicada recentemente^38^. Em cerca de 40.000 pacientes avaliados (variação da média de idade de 67 a 77 anos, 48 a 54% dos pacientes do sexo feminino e insuficiência cardíaca congestiva como o principal motivo da admissão hospitalar), foi demonstrada a redução de eventos de TEV sintomático ou de morte relacionada ao TEV em comparação à profilaxia padrão (0,8% vs. 1,2%; risco relativo [RR]: 0,61; IC95%: 0,44–0,83; p = 0,002). No entanto, houve aumento do risco de hemorragia maior ou fatal (0,6% vs. 0,3%; RR: 2,04; IC95%: 1,42–2,91; p < 0,001). A análise demonstrou que o número necessário para prevenir um evento sintomático de TEV (NNT, do inglês *number needed to treat*) ou morte relacionada ao TEV foi de 250, enquanto o número necessário para causar um evento hemorrágico principal ou fatal (NNH, do inglês *number needed to harm*) foi de 333^38^. São reconhecidas as diversas limitações dos estudos avaliados, como a variabilidade dos critérios de inclusão e da duração da farmacoprofilaxia estendida, além dos protocolos de diagnóstico de TEV utilizados^38^. O resultado dessa metanálise traz uma importante reflexão sobre a necessidade de equilibrar a decisão entre eficácia e benefícios desejáveis da profilaxia estendida com a segurança e danos potenciais de seu uso, uma vez que o valor do NNT para evitar um evento sintomático (250) é muito próximo ao valor do NNH (333) necessário para causar um sangramento maior^38^.

## FARMACOPROFILAXIA ESTENDIDA EM PACIENTES CIRÚRGICOS

Para a avaliação da farmacoprofilaxia para TEV em pacientes cirúrgicos, foram propostas recomendações quanto ao tempo de duração baseadas na pontuação do escore de Caprini^23^^,^ 39 ^,^ 40, originalmente validado para cirurgia geral abdominal e pélvica, vascular, bariátrica e plástica reconstrutora. Naqueles pacientes classificados como de risco moderado (escore de Caprini 3 ou 4), sugere-se a farmacoprofilaxia exclusivamente hospitalar. A farmacoprofilaxia por 7 a 10 dias está recomendada para pacientes de alto risco (escore de Caprini 5 a 8). Naqueles com risco altíssimo (escore de Caprini > 8), deve ser orientada a profilaxia prolongada por 30 dias, salvo contraindicações^39^^,^ 40 (Tabela 2). Estão incluídos no grupo de altíssimo risco os indivíduos submetidos a cirurgias ortopédicas maiores, como artroplastia eletiva total de quadril (ATQ) ou de joelho (ATJ) e cirurgia de fratura pélvica ou de quadril, assim como trauma grave, lesão medular e cirurgia oncológica abdominal ou pélvica extensas^16^^,^ 17.

**Tabela 2:** Recomendações de regimes e tempo de profilaxia de tromboembolismo venoso em pacientes cirúrgicos com base no escore de Caprini.

**Escore de Caprini**	**Risco categorizado**	**Recomendação de profilaxia**	**Duração da profilaxia**
0	Muito baixo	Deambulação precoce e frequente isolada ou de acordo com a avaliação da equipe cirúrgica: CPI ou baixa dose de HNF ou HBPM	Durante a hospitalização
1-2	Baixo	CPI ou baixa dose de HNF ou HBPM	Durante a hospitalização
3-4	Moderado	CPI e baixa dose de HNF ou HBPM	Durante a hospitalização
5-8	Alto	CPI e baixa dose de HNF ou HBPM	7 a 10 dias no total
> 8	Muito alto	CPI e baixa dose de HNF ou HBPM	30 dias no total

HNF = heparina não fracionada; HBPM = heparina de baixo peso molecular; CPI = compressão pneumática intermitente.

Adaptada de: Cassidy et al.^39^.

Todos os pacientes com neoplasia maligna submetidos a grandes intervenções cirúrgicas devem receber farmacoprofilaxia com HNF ou HBPM, a menos que contraindicado por causa de sangramento ativo ou alto risco potencial de sangramento^17^^,^ 18. A profilaxia estendida com HBPM por até 4 semanas no pós-operatório está recomendada para pacientes submetidos à cirurgia oncológica abdominal ou pélvica maior, aberta ou laparoscópica^17^. A última diretriz da *American Society of Clinical Oncology* recomenda o uso de HBPM por até 4 semanas no pós-operatório de grandes cirurgias abdominais ou pélvicas, em pacientes oncológicos que apresentam características de alto risco de TEV: mobilidade restrita, obesidade, TEV prévio ou com fatores de risco adicionais. Em situações de menor risco, a decisão deve ser individualizada^18^.

Outros pacientes cirúrgicos para os quais a profilaxia estendida está bem estabelecida são aqueles submetidos a ATJ ou ATQ eletivas e os submetidos a cirurgia para correção de fratura proximal do fêmur (FPF)^16^^,^ 41. Recomenda-se o uso de farmacoprofilaxia por no mínimo 10 a 14 dias para ATJ e 28 a 35 dias para ATQ com HBPM, HNF, fondaparinux, apixabana, dabigatrana, rivaroxabana, varfarina ou ácido acetilsalicílico (AAS), ou ainda profilaxia mecânica com compressão pneumática intermitente (CPI) para aqueles com alto risco de sangramento^16^. Para pacientes submetidos à ATJ, a nona ACCP recomenda estender a profilaxia por até 35 dias a partir da cirurgia, em comparação ao uso limitado por 10 a 14 dias. Nos pacientes submetidos à correção de FPF, o tempo de profilaxia deve ser de 28 a 35 dias, devendo-se utilizar HNF, HBPM ou fondaparinux, uma vez que os DOACs não foram aprovados para essa indicação^16^^,^ 41. Para pacientes submetidos a ATQ, ATJ ou cirurgia de FPF, sugere-se o uso combinado de profilaxia farmacológica e mecânica com CPI de membros inferiores durante a hospitalização^16^.

## ESTRATÉGIAS PARA OTIMIZAR A ADESÃO À PROFILAXIA DE TEV NA ALTA HOSPITALAR

Embora o risco de TEV após a alta hospitalar seja amplamente reconhecido entre pacientes de alto risco, estudos demonstram que a profilaxia estendida ainda é subutilizada^42^^,^ 43. A identificação de barreiras que limitem a adesão a essa prática de segurança e a implementação de estratégias facilitadoras devem representar um dos focos de atuação das instituições de saúde.

Considerando-se que o tempo de hospitalização é frequentemente inferior ao tempo total recomendado de uso dos anticoagulantes, as orientações de tromboprofilaxia devem ser ajustadas às estratégias de desospitalização precoce para evitar os eventos após a alta e as readmissões por TEV^1^. Assim, o planejamento da alta hospitalar é uma etapa essencial para assegurar a transição dos cuidados e a eficácia global da tromboprofilaxia nos diferentes cenários clínicos^1^^,^ 41. Algumas estratégias podem ser incorporadas na prática assistencial para manter a qualidade e a segurança na prevenção do TEV (Tabela 3).

**Tabela 3:** Ações facilitadoras da implementação da alta segura na prevenção do tromboembolismo venoso (TEV).

Protocolo institucional para profilaxia de TEV que inclua recomendações de profilaxia estendida
Utilização regular de MARs para TEV
Estratégias de tecnologia que incorporem alertas eletrônicos aos prontuários na admissão e no momento da desospitalização
Planos terapêuticos que contemplem as principais linhas de cuidados institucionais
Busca ativa de pacientes elegíveis à farmacoprofilaxia estendida na internação
Utilização de *checklist* para o planejamento da alta
Elaboração de sumários multidisciplinares de alta
Treinamento da equipe multidisciplinar para fornecer orientações de alta
Elaboração de materiais educativos para pacientes e familiares
Criação de comissões de desospitalização

MARs = modelos de avaliação de risco.

Adaptada de: Barkoudah^1^.

## CONCLUSÃO

A elevada incidência de eventos de TEV após a desospitalização reforça a necessidade da avaliação individualizada de risco de TEV dos pacientes clínicos e cirúrgicos na alta. A redução dos eventos tardios depende do reconhecimento de grupos de pacientes com potencial benefício da farmacoprofilaxia por tempo prolongado, sem aumento do risco de sangramento^38^. Essas definições são mais claras para pacientes cirúrgicos ortopédicos e oncológicos de alto risco. Em pacientes clínicos, a extensão da profilaxia confere benefício à prevenção de eventos^30^^-^ 34, mas aumenta as taxas de sangramento maior^30^^-^ 32 e de sangramento NMCR^32^^-^ 34, mesmo em populações em que foram aplicados critérios mais rígidos de exclusão de pacientes com maior risco hemorrágico. A utilização de múltiplas estratégias para aumentar a adesão ao protocolo de tromboprofilaxia e a mensuração dos resultados com propostas de melhorias são as principais ações institucionais para garantir a adequada proteção dos pacientes.
